# Compatibility of metal additive manufactured tungsten collimator for SPECT/MRI integration

**DOI:** 10.1186/2197-7364-2-S1-A52

**Published:** 2015-05-18

**Authors:** Amine M Samudi, Karen Van Audenhaege, Gunter Vermeeren, Luc Martens, Roel Van Holen, Wout Joseph

**Affiliations:** 1 INTEC, Ghent University/iMinds, Ghent, Belgium; 2 ELIS, Ghent University/iMinds, Gent, Belgium

We optimized the MR-compatibility of a novel tungsten collimator, produced with metal additive manufacturing that is part of a microSPECT insert for a preclinical SPECT/MRI scanner. We characterized the current density due to the gradient field and adapted the collimators by smart design to reduce the induced eddy currents. The z-gradient coil and the collimator were modeled with SEMCAD. The gradient strength was 510 mT/m, the gradient efficiency was about 3.4 mT/m/A. The setup was simulated with a working frequency of 10 kHz. The system consists of 7 identical collimators and digital silicon photomultipliers assembled in a ring. We evaluated the global reduction in current density J (reduction) based on the sum of all current densities in the collimator. We applied the following optimizations on the collimator: 1. We reduced the excessive material in the flanges. 2. We applied horizontal slits of 2 mm in the collimator surface. 3. We reduced material in the core; the photons are attenuated before they reach the core. The collimator will need a supporting structure. 4. The supporting structure can be avoided by using two vertical slits in the middle of the collimator. 5. We used a Z-shaped slit instead of the vertical slit. Results of simulations show that smaller flanges reduce the current density with 23%. The horizontal slits reduce the eddy currents with 6%. Using less material in the core or applying vertical slits results in the same reduction of current density. However, the vertical slits are cheaper because a hollow collimator requires supporting structures during production. Both can be combined if z-shaped slits are used to prevent attenuation problems. The reduction is then 27%. Finally, when all previous adaptations are combined, the reduction in eddy currents is about 56.3%.

